# *Hibiscus sabdariffa* Extract Protects HaCaT Cells against Phenanthrene-Induced Toxicity through the Regulation of Constitutive Androstane Receptor/Pregnane X Receptor Pathway

**DOI:** 10.3390/nu14183829

**Published:** 2022-09-16

**Authors:** Dicson Sheeja Malar, Mani Iyer Prasanth, Kanika Verma, Anchalee Prasansuklab, Tewin Tencomnao

**Affiliations:** 1 Natural Products for Neuroprotection and Anti-Ageing Research Unit, Chulalongkorn University, Bangkok 10330, Thailand; 2 Department of Clinical Chemistry, Faculty of Allied Health Sciences, Chulalongkorn University, Bangkok 10330, Thailand; 3 Department of Parasite-Host Biology, ICMR-National Institute of Malaria Research (NIMR), New Delhi 110077, India; 4 College of Public Health Sciences, Chulalongkorn University, Bangkok 10330, Thailand

**Keywords:** phenanthrene, constitutive androstane receptor, pregnane X receptor, oxidative stress, CYP1A1, keratinocytes

## Abstract

Phenanthrene (Phe) exposure is associated with skin ageing, cardiotoxicity and developmental defects. Here, we investigated the mode of Phe toxicity in human keratinocytes (HaCaT cells) and the attenuation of toxicity on pre-treatment (6 h) with ethanol extract of *Hibiscus sabdariffa* calyxes (HS). Cell viability, reactive oxygen species (ROS) generation, mitochondrial membrane potential (ΔΨm) alteration, changes in the transcriptional activity of selected genes involved in phase I and II metabolism, antioxidant response and gluconeogenesis, western blot and docking studies were performed to determine the protective effect of HS against Phe. Phe (250 μM) induced cytotoxicity in HaCaT cells through AhR-independent, CAR/PXR/RXR-mediated activation of CYP1A1 and the subsequent alterations in phase I and II metabolism genes. Further, CYP1A1 activation by Phe induced ROS generation, reduced ΔΨm and modulated antioxidant response, phase II metabolism and gluconeogenesis-related gene expression. However, pre-treatment with HS extract restored the pathological changes observed upon Phe exposure through CYP1A1 inhibition. Docking studies showed the site-specific activation of PXR and CAR by Phe and inhibition of CYP1A1 and CYP3A4 by the bioactive compounds of HS similar to that of the positive controls tested. Our results conclude that HS extract can attenuate Phe-induced toxicity in HaCaT cells through CAR/PXR/RXR mediated inhibition of CYP1A1.

## 1. Introduction

The World Health Organization (WHO) describes air pollution as a prominent risk factor accounting for one in nine deaths worldwide [1]. Human exposure to particulate matter (PM) less than 2.5 μm (PM2.5) was reported to have several adverse effects [2]. Polycyclic aromatic hydrocarbons (PAHs) are pervasive toxic air pollutants and a key organic class of compounds in PM2.5, which are formed from the partial combustion of fossil fuel, exhaust from vehicles and industrial processes [3]. The generation and exposure of PAHs in humans, animals and aquatic organisms is a huge concern, as they are highly mutagenic, teratogenic and carcinogenic [4]. Most of the PAHs exert their toxicity/carcinogenicity through the activation of the aryl hydrocarbon receptor (AhR) and the subsequent transactivation of downstream target genes, including cytochrome P450s (CYPs) and other xenobiotic response elements (XRE) [5]. Some of the PAHs in their native form may act as weak carcinogens, but the action of xenobiotic metabolizing enzymes over them generates mutagenic metabolites that can form covalent DNA adducts to initiate carcinogenic processes [6]. In addition, the non-carcinogenic PAHs could activate the nuclear receptors, including the constitutive androstane receptor (CAR) and pregnane X receptor (PXR), in association with the retinoid receptor (RXR) or tamper intracellular signalling mechanisms to contribute toward PAH-mediated toxicity [7,8,9,10]. 

Of the several hundred, the Environmental Protection Agency (EPA) has characterized 16 PAHs as high-priority pollutants, including phenanthrene (Phe), which serves as a biomarker for PAH exposure in humans [11]. The Phe is of a low molecular weight PAH with bay- and K-regions that are needed to form highly reactive epoxides upon metabolism [12]. Human exposure to Phe occurs via diet, inhalation and skin absorption, of which dietary intake accounts for the main route of entry [13,14,15]. The concentration of Phe (84.6 to 191 μg/kg) was observed to be high in fish [16]. The human milk and urine of infants were also detected with Phe in the concentration of 24.1 ng/g of milk fat and 0.03 mg/L, respectively [17,18]. Although scientific evidence concerning the carcinogenicity and mutagenicity of Phe is lacking, there exist several reports on its bioaccumulation and toxicity in various cellular models, aquatic organisms and soil nematodes, indicating the environmental and health impact of the pollutant [19,20]. Pioneering studies report that Phe exposure can lead to cardiotoxicity, neurotoxicity, and developmental and reproductive defects in experimental model systems [21,22,23,24]. Moreover, Phe bioaccumulation can also generate reactive oxygen species (ROS) and modulates the activity of antioxidant defence enzymes, causing oxidative stress and pathogenesis [25]. A study by Sarkar et al. (2017) reports a direct correlation between PAH exposure and alteration in antioxidant activity and DNA damage, indicating the need for antioxidant system modulators for protection against toxicity [26]. In addition, chronic PAH exposure to the skin could result in skin damage, including wrinkle formation, pigmentation, and skin cancer [27,28]. A study by Luo et al. (2020) has reported that the total dermal penetration and absorption rate of Phe by skin cells are high compared to pyrene and benzo(a)pyrene [29].

Plant extracts are of immense interest because of their low toxic profile as well as the combinatorial/synergistic actions of their metabolites to combat pathological conditions. Several studies have reported that the consumption of fruits, vegetables or treatment of extracts in cellular models can modulate the metabolism of carcinogens/drugs, inhibit the CYP metabolizing enzymes and convert them into inactive metabolites [30,31,32,33,34,35,36]. Recently, topical application of cosmetics containing herbal extracts as well as their dietary supplementation has been in demand due to their therapeutic application and safety [37]. Dietary supplementation of four standardized herbal extracts (Zeropollution^®^) was reported to protect against skin ageing by inhibiting pollutant-induced oxidative stress [38]. *Hibiscus sabdariffa* (Roselle) belongs to the family Malvaceae and is widely distributed throughout tropical and subtropical regions. The leaves of the plant are consumed as vegetables, and the calyxes are used for the preparation of beverages. Previous studies have reported on the various therapeutic activity of the plant both in in vitro and in vivo model systems. The topical application of the roselle extract ointment was reported to induce wound healing by inhibiting inflammatory pathways [39]. Some of the bioactive compounds from *H. sabdariffa* have been regarded as potential cosmetic applicants, which can exhibit anti-melanin and depigmentation properties [40,41,42]. *H. sabdariffa* extract has been previously reported to show an anti-ageing effect [43,44]. Also, a previous study from our lab has identified the presence of phytochemicals including epigallocatechin 3-*O*-(3-*O*-methylgallate), pelargonidin 3-*O*-glucoside, allo-aromadendrene, anisocoumarin H and *N*-Feruloyltyramine [45]. The current study is focused on exploring the protective effect of ethanol extract of *H. sabdariffa* calyxes (HS) against Phe exposure in immortalized human keratinocytes HaCaT cells, which are the initial target of environmental pollutant exposure.

## 2. Materials and Methods

### 2.1. Chemicals

All the cell culture mediums used in the study were procured from HyClone, Logan, UT, USA. The Human skin keratinocyte HaCaT cells were procured from Cell Line Services (CLS, Eppelheim, Germany). Monoclonal mouse primary antibodies (anti-AhR, anti-CYP1A1, anti-Bcl-2, anti-Bax) and Monoclonal rabbit primary antibodies (anti-β-actin) were obtained from Santa Cruz Biotechnology, Dallas, TX, USA and Cell Signaling Technology, Danvers, MA, USA respectively. Phenanthrene, Anti-rabbit/mouse IgG- HRP linked secondary antibodies were purchased from Sigma Aldrich, Burlington, MA, USA. ECL Western blotting substrate was obtained from GE Healthcare, USA.

### 2.2. Plant Collection and Extraction

Plant collection, authentication, extraction using ethanol, stock preparation and storage were done as reported earlier [44]. The working solution was prepared in Dulbecco’s modified Eagle medium (DMEM), sterile filtered with a 0.2 µm pore size syringe filter and used for the experimental analysis. 

### 2.3. Cell Viability Assay

The HaCaT cells (2 × 10^4^ cells) were seeded in a 96-well plate in complete medium (DMEM, 10% Fetal bovine serum, 1X Penicillin-Streptomycin) and allowed to grow overnight. The cells were treated with various doses of Phe (50–250 μM) and HS extract (20–100 μg/mL) for 24 h and subjected to an MTT assay to find the toxic dose of Phe and the non-toxic dosage range of extract. To evaluate the protective effect of HS extract, HaCaT cells were pre-treated (6 h) with HS extract (20–100 μg/mL) followed by Phe (250 μM) and incubated for 24 h. After incubation, cells were subjected to the MTT assay, and the absorbance was measured at 540 nm [44].

### 2.4. Measurement of Reactive Oxygen Species (ROS) Level and Mitochondrial Membrane Potential (*ΔΨm*)

The generation of ROS and alteration in ΔΨm upon Phe treatment was measured quantitatively. HaCaT cells (2 × 10^5^ cells) were grown in a 96-well black plate. Cells were pre-treated with HS extract (50, 60 μg/mL) for 6 h and then with Phe (250 μM). After treatment, cells were washed thrice with PBS, and 10 µM DCFH-DA (for ROS) or 5 μM Rhodamine 123 (Rh 123) (for ΔΨm) was added and incubated for 30 min in a CO_2_ incubator at 37 °C. Cells were processed according to the standard protocols [44], and the fluorescence was measured using EnSpire Multimode Plate Reader (PerkinElmer, Waltham, MA, USA) with excitation and emission of 480 and 535 nm, respectively. The results of fluorescence intensity were represented as a % control. For qualitative analysis of ROS, HaCaT cells were grown in coverslips, and the treatment was done as mentioned earlier. After 24 h, cells were washed and incubated with 10 µM DCFH-DA for 30 min. After incubation, cells were washed and imaged under a fluorescence microscope (Ziess Axio Observer A1).

### 2.5. Real-Time PCR Analysis

After treatment of cells, as mentioned earlier, the cells were collected, and total RNA was extracted using the standard Trizol procedure and quantified. The extracted RNA (1000 ng) was reverse-transcribed using the Maxime^TM^ RT Premix kit with oligo(dT)15 primer (Intron biotech, South Korea), and then real-time PCR (qPCR) analysis was performed with the RealMOD Green W^2^ 2x qPCR mix (Intron biotech) with gene-specific primers (Table 1), and the data were normalized to the endogenous control (GAPDH) [44].

### 2.6. Western Blot Analysis

The cells, after treatment, were collected and lysed with NP-40 lysis buffer. Separation of proteins (50 μg) was done on 12% SDS gels and subsequently transferred to polyvinylidene difluoride (PVDF) membranes. Overnight blocking of the membrane was done in 5% skim milk, which was followed by 6 h incubation with respective primary antibodies (AhR (1:1000), CYP1A1 (1:1000), Bcl-2 (1:1000), Bax (1:1000), β-actin (1:5000)). Subsequently, the membranes were incubated for one h with anti-mouse/rabbit IgG-HRP linked secondary antibody (1:10,000). The bands were developed with ECL Western blot detection reagent, imaged and further quantified with ImageJ software [44]. 

### 2.7. Molecular Docking Analysis

The structures of the target proteins, orphan nuclear receptor NR1I3/CAR (PDB ID: 1XVP), PXR (PDB ID: 6DUP), CYP1A1 (PDB ID: 4I8V), CYP3A4 (PDB ID: 5VCE) were retrieved from PDB database. The water molecules, other ligands and cofactors were removed from the protein structures, followed by energy minimization using Swiss-PDBViewer software [46]. Molecular docking was performed with the compounds identified previously from HS through LC-MS/MS analysis [45]. The ligand structures were downloaded from the PubChem database [47]. To gain insight into the possible mode of action of the ligands and the possible interactions with protein, docking studies were performed using DockThor, which uses a grid-based docking method to compute different modes of ligand binding on the protein. We have performed site-specific docking with the standard mode feature of the tool [48,49]. Docking calculations were conducted for each protein with the centre (average of the X, Y, and Z coordinates) and grid size (20 × 20 × 20) to define the grid box. The position of the grid box’s centre in each protein is represented in Table 2. The discretization was kept at 0.25 Å. Later the results were compared with the respective positive controls for each protein. 

### 2.8. Statistical Analysis

All the experiments were done in triplicates and expressed as Mean ± SD. One-way ANOVA (SPSS 17, SPSS Inc., Chicago, IL, USA) followed by Tukey’s post hoc test was performed to compare control vs treated and *p* < 0.05 was considered significant. 

## 3. Results

### 3.1. HS Extract Pre-Treatment Protects HaCaT Cells from Phe Induced Cell Death

**Figure 1:**
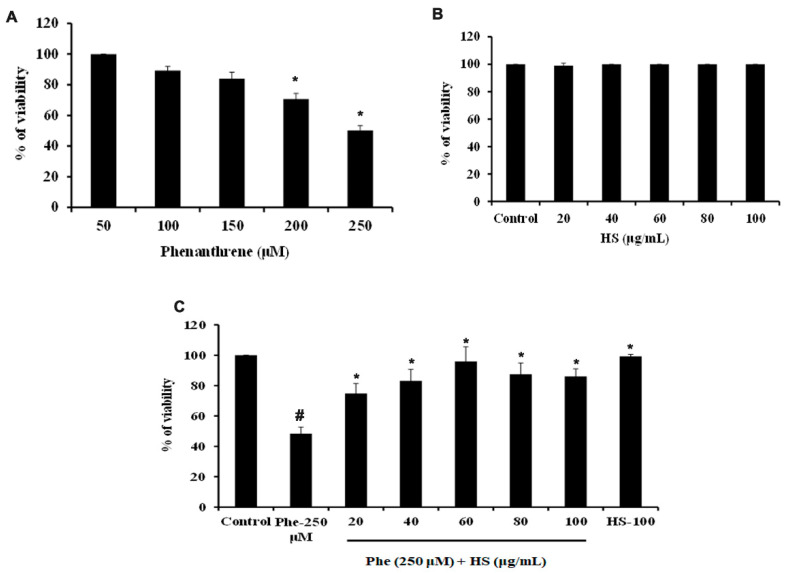
(**A**) Phe induced cytotoxicity in HaCaT cells in a dose-dependent manner (**B**) Effect of HS extract on HaCaT cells (**C**) Pre-treatment (6 h) of HS extract protected HaCaT cells from Phe (250 μM) induced toxicity in a concentration-dependent manner (*n* = 3; Significance at *p* < 0.05; # indicates Control vs Phe; * indicates Phe vs. HS pre-treatment; HS—*Hibiscus sabdariffa*; Phe—Phenanthrene).

Treatment of Phe to HaCaT cells induced cell death in a dose-dependent manner at 24 h. At lower doses (below 100 μM), Phe did not induce cell death, whereas a significant reduction in cell viability was observed from 200 μM concentration. The dose of 250 μM, which exhibited 50% cell death, was fixed as the toxic concentration and used for further studies (Figure 1A). Likewise, a varying range of concentrations of HS extract (20–100 μg/mL) was checked for toxicity in HaCaT cells. HS extract did not exhibit toxicity in the tested range for 24 h (Figure 1B), indicating a non-toxic profile of the extract. Further, the non-toxic range of concentrations of extract were analyzed for their protective effect against Phe-induced cell death. Pre-treatment with HS extract for 6 h showed a significant (*p* < 0.05) increase in cell viability until 60 μg/mL, after which a decrease in the trend was found for 80 and 100 μg/mL. However, the HS alone treatment (100 μg/mL) group retained the cell viability similar to the control (Figure 1C). 

**Figure 2:**
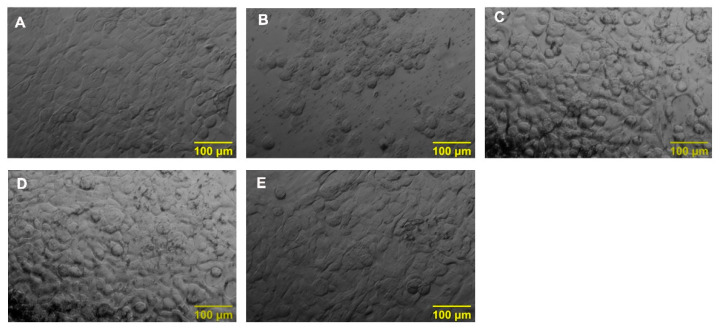
Morphological assessment of HaCaT cells on exposure with Phe and pre-treatment with HS extract (**A**) Control (**B**) Phe (250 μM) (**C**) Phe + HS50 (**D**) Phe + HS60 (**E**) HS60 (scale bar—100 μm).

The morphological changes in HaCaT cells upon Phe exposure and restoration of cell viability on pre-treatment with HS were shown in Figure 2. The control group showed cobblestone morphology (Figure 2A), which is typical for HaCaT cells, whereas Phe exposure altered the morphology and showed rounded and distorted structures (Figure 2B). However, pre-treatment with HS extract restored the cell morphology indicating the protective effect (Figure 2C–E). From the observed results, the doses of 50 and 60 μg/mL (pre-treatment) were fixed for further experiments.

### 3.2. Phe Induced Toxicity through the AhR-Independent Activation of CYP1A1

**Figure 3:**
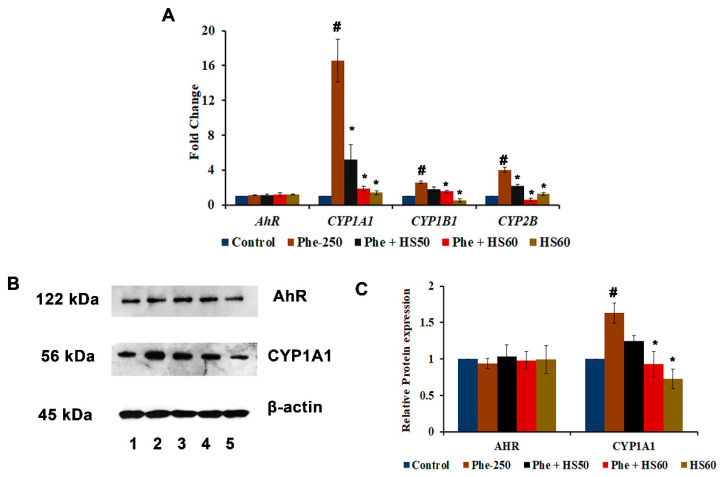
(**A**) Transcriptional regulation of Phase I metabolism genes by HS upon Phe toxicity (**B**) Phe-induced CYP1A1 protein expression through AhR independent mechanism, whereas HS pre-treatment reversed the effect [Lane-1: Control; 2: Phe (250 μM); 3: Phe + HS50; 4: Phe + HS60; 5: HS60] (**C**) Quantification for western blot analysis (*n* = 3; Significance at *p* < 0.05; # indicates Control vs. Phe; * indicates Phe vs. HS pre-treatment; HS—*Hibiscus sabdariffa*; Phe—Phenanthrene).

As most of the PAHs exert their toxicity through the AhR-dependent activation of CYPs, the expression of AhR and CYPs were studied upon Phe-induced toxicity in HaCaT cells. Firstly, the transcriptional activation of *AhR* and various *CYPs,* including *CYP1A1*, *CYP1B1*, and *CYP2B,* was determined. Significant (*p* < 0.05) increases in the expression of *CYP1A1* (16.5-fold), *CYP1B1* (2.6-fold), and *CYP2B* (4.0-fold) were observed upon Phe treatment when compared to the control, whereas no significant change in the expression of *AhR* was observed (Figure 3A). The results indicate that Phe exerts toxicity through *AhR*-independent activation of *CYP1A1*. However, the pre-treatment (6 h) with 50, 60 μg/mL HS extract showed a significant (*p* < 0.05) reduction in the expression of *CYPs* (Figure 3A). Further, the changes in the protein expression of AhR and CYP1A1 were studied. The results corroborate with the gene expression studies, where a significant upregulation (*p* < 0.05) of CYP1A1 was observed with no significant change in AhR expression upon Phe-induced toxicity, and HS treatment could significantly restore the changes similar to the control (Figure 3B,C). The results indicate that Phe exerts toxicity through AhR-independent activation of CYP1A1 and HS pre-treatment exhibits a protective effect through the inhibition of CYP1A1.

**Figure 4:**
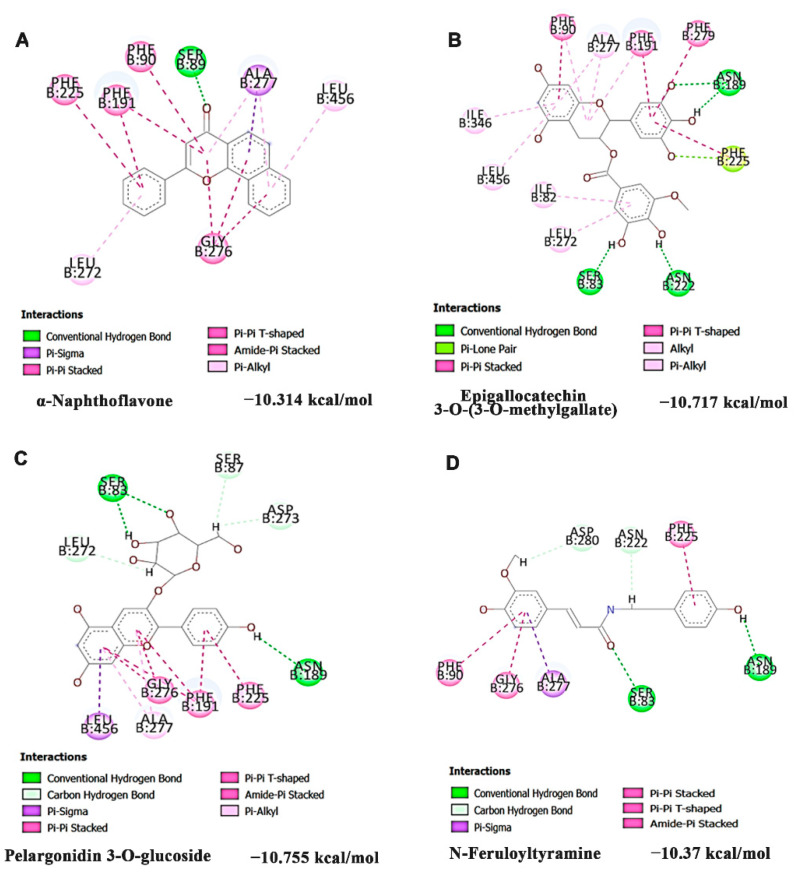
Bioactive constituents of HS extract inhibited CYP1A1 in a site-specific manner as identified by docking analysis (**A**) α-Naphthoflavone (antagonist) (**B**) Epigallocatechin 3-*O*-(3-*O*-methyl gallate (**C**) Pelargonidin 3-*O*-glucoside (**D**) *N*-Feruloyltyramine.

Additionally, the inhibitory potential of the compounds of HS extract identified through LC-MS/MS analysis [45] against CYP1A1 was performed through docking analysis with the reference compound α-Naphthoflavone (antagonist). The interaction of the top three hits along with α-Naphthoflavone against CYP1A1 was represented in Figure 4. The reference compound α-Naphthoflavone exhibited a docking score of −10.314 kcal/mol and showed interaction with the residues SER89, PHE90, PHE191, PHE225, LEU272, GLY276, ALA277, and LEU456 (Figure 4A). The compounds of HS extract, including Epigallocatechin 3-*O*-(3-*O*-methylgallate) (−10.717 kcal/mol), Pelargonidin 3-*O*-glucoside (−10.581 kcal/mol), and *N*-Feruloyltyramine (−10.37 kcal/mol) showed higher docking score and interacted in the same binding pocket as that of the reference compound α-Naphthoflavone (Figure 4B–D).

### 3.3. Phe Induced CYP1A1 Activity Was Mediated through the Activation of CAR/PXR/RXR Nuclear Receptors

**Figure 5:**
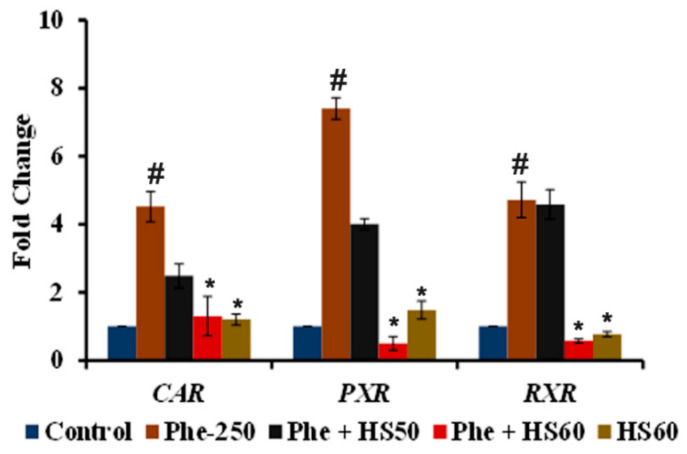
HS extract inhibited Phe-induced activation of the nuclear receptors CAR, PXR and RXR to attenuate CYP1A1 expression (*n* = 3; Significance at *p* < 0.05; # indicates Control vs. Phe; * indicates Phe vs. HS pre-treatment).

To identify the mechanism through which CYP1A1 is activated upon Phe toxicity, the gene expression of the xenobiotic sensing receptors *CAR, PXR, and RXR* were analyzed. A significant (*p* < 0.05) upregulation in the expression of the nuclear receptors *CAR* (4.5-fold), *PXR* (7.4-fold), and *RXR* (4.7-fold) were observed upon Phe treatment (Figure 5). However, a significant reversal in trend was observed upon treatment with HS (60 μg/mL). The results indicate that the Phe-induced CYP1A1 activity was mediated through the *CAR/PXR/RXR* pathway.

**Figure 6:**
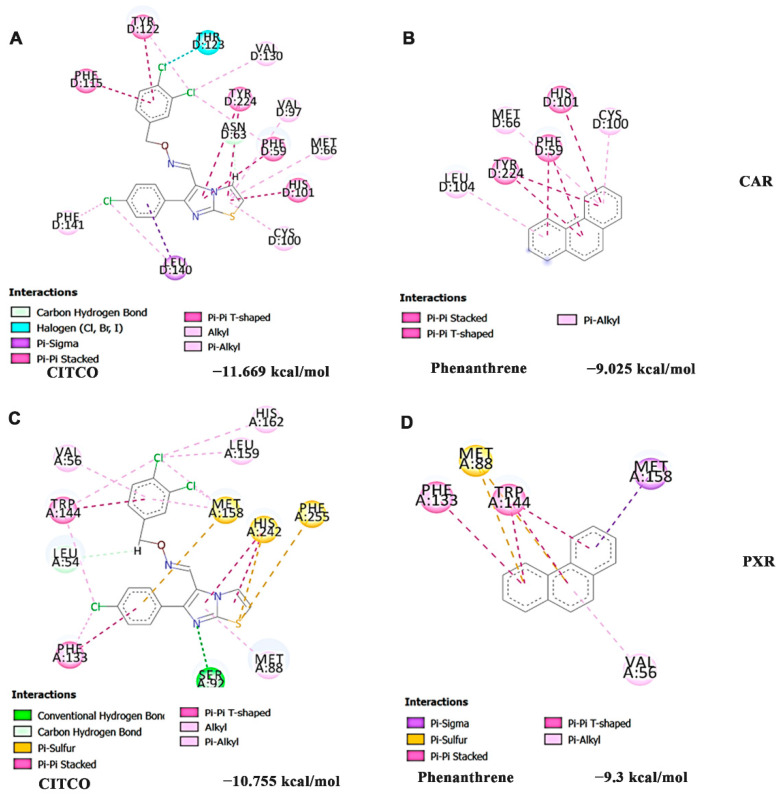
Site-specific activation of CAR and PXR by Phe as identified by docking analysis in comparison with the agonist CITCO (**A**) CITCO (agonist) against CAR (**B**) Phenanthrene against CAR (**C**) CITCO (agonist) against PXR (**D**) Phenanthrene against PXR (CAR—Constitutive androstane receptor; PXR—Pregnane X receptor; CITCO—6-(4-Chlorophenyl)imidazo[2,1-B][1,3]thiazole-5-carbaldehyde O-(3,4-dichlorobenzyl)oxime).

In addition, in silico docking studies were performed with Phe and the agonist CITCO (which can activate both the receptors) against the nuclear receptors CAR and PXR. The docking was performed for Phe on the same binding site as CITCO. The docking results show that Phe could bind and interact to the same binding site as that of CITCO with both nuclear receptors (Figure 6A–D). In the case of CITCO against CAR, the compound could interact with MET66, CYS100, HIS101, and TYR204, similar to that of Phe, along with other residues including VAL97, PHE115, VAL130 and LEU140 through Pi-Sigma, Pi-Pi T-shaped, Pi-Pi stacked and alkyl bonds with a higher binding score of −11.669 kcal/mol than Phe (−9.025 kcal/mol) (Figure 6A,B). Likewise, against PXR, CITCO interacted with VAL56, MET88, PHE133, and TRP144, similar to Phe, along with other residues SER92, MET158, LEU159, HIS162, HIS242 and PHE255 through conventional hydrogen, Pi-Sulfur, Pi-Pi T-shaped, Pi-Pi stacked and alkyl bonds with higher docking score of −10.755 kcal/mol compared to Phe (−9.3 kcal/mol) (Figure 6C,D). Although the docking score of Phe is less when compared to the reference compound CITCO, it should be noted that the site of interaction is the same, and the difference in the binding scores is relatively small; hence Phe can still bind and activate the respective targets.

**Figure 7:**
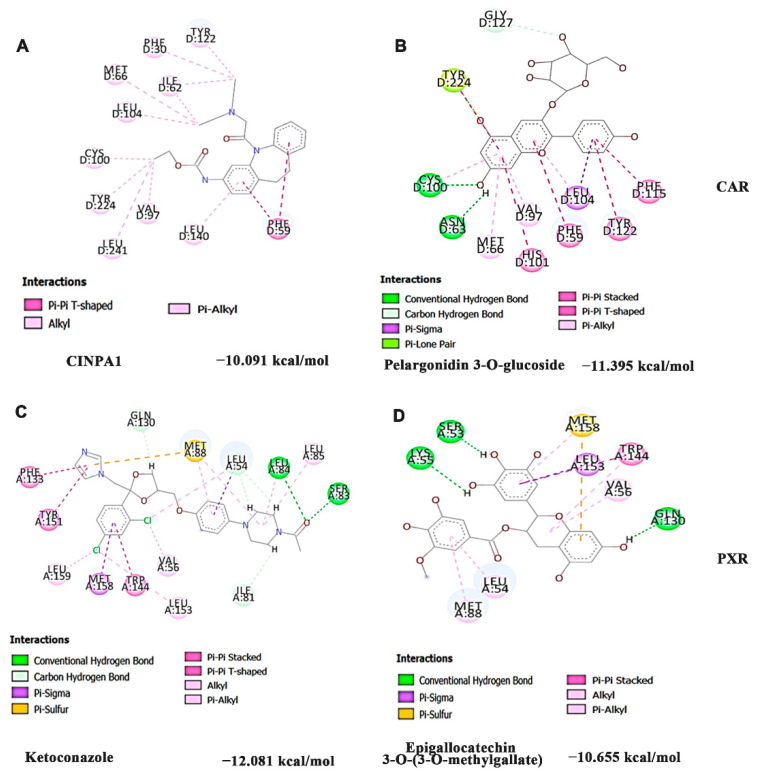
Site-specific inhibition of CAR and PXR by compounds (top hit) of HS extract in comparison with the respective antagonists (**A**) CINPA1 (inhibitor) against CAR (**B**) pelargonidin 3-*O*-glucoside against CAR (**C**) Ketoconazole (inhibitor) against PXR (**D**) Epigallocatechin 3-*O*-(3-*O*-methyl gallate against PXR (CAR—Constitutive androstane receptor; PXR—Pregnane X receptor; CINPA1—Ethyl [5-[(diethylamino)acetyl]-10,11-dihydro-5H-dibenz[b,f]azepin-3-yl]carbamate).

Further, the inhibitory effect of the compounds identified from HS extract against CAR and PXR was evaluated by molecular docking analysis with CINPA1 (CAR) and ketoconazole (PXR) as positive controls. Docking was performed at the same site where Phe was found to bind and activate the receptors. The interaction of positive control CINPA1 and top hit pelargonidin 3-*O*-glucoside against CAR was represented in Figure 7A,B, and the interaction of positive control ketoconazole and the top hit Epigallocatechin 3-*O*-(3-*O*-methyl gallate against PXR was represented in Figure 7C,D. Against CAR, the positive control CINPA1 (−10.091 kcal/mol) and the compounds of HS including pelargonidin 3-*O*-glucoside (−11.395 kcal/mol), allo aromadendrene (−11.212 kcal/mol), and Epigallocatechin 3-*O*-(3-*O*-methyl gallate (−10.913 kcal/mol) showed similar interactions as Phe with the amino acids PHE59, MET66, CYS100, HIS101, LEU104, and TYR224. Likewise, against PXR, the positive control ketoconazole showed a higher docking score of −12.081 kcal/mol, followed by Epigallocatechin 3-*O*-(3-*O*-methyl gallate (−10.655 kcal/mol), pelargonidin 3-*O*-glucoside (−10.29 kcal/mol), and anisocoumarin H (−9.797 kcal/mol). The compounds showed interactions with amino acids, including VAL56, MET88, PHE133, TRP144 and MET158, which are similar to that of Phe against PXR. The results indicate that the compounds of HS can block the binding of Phe on the active site of both the receptors and inhibit their activation to mediate the protective effect.

### 3.4. HS Extract Restored the Expression of CAR/PXR Target Genes Encoding Phase-I and II Drug Metabolizing Enzymes to Protect against Phe Toxicity

**Figure 8:**
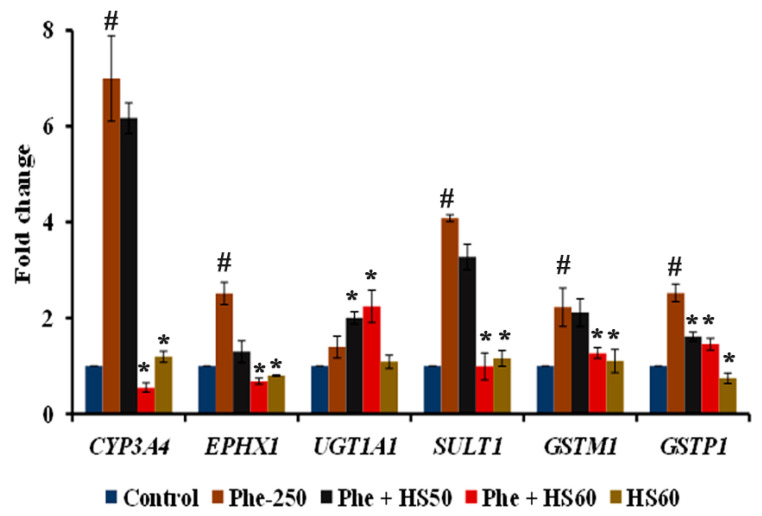
Transcriptional regulation of *CAR/PXR* pathway regulated genes involved in phase I and II metabolism by HS against Phe toxicity (*n* = 3; Significance at *p* < 0.05; # indicates Control vs. Phe; * indicates Phe vs. HS pre-treatment).

The modulation of some of the target genes of *CAR/PXR*, which encodes Phase-I and II drug metabolizing enzymes by Phe, was analyzed. The expression of *CYP3A4* (6.9-fold ± 0.89), a prototypical Phase-I target of CAR/PXR, was found to be significantly (*p* < 0.05) increased, further indicating the activation of the CAR/PXR pathway by Phe. Likewise, an increased expression of Phase-II target genes by Phe was also observed. For instance, *EPHX1* (2.51 ± 0.23-fold), *UGT1A1* (1.39 ± 0.22-fold), *SULT1* (4.1 ± 0.07-fold), *GSTM1* (2.22 ± 0.4-fold) and *GSTP1* (2.52 ± 0.18-fold) showed a significant (*p* < 0.05) increase in expression upon Phe treatment when compared to the control. A significant reversal in the effect caused by Phe was observed with HS (60 μg/mL) pre-treatment except for *UGT1A1*. The pre-treatment of HaCaT cells with HS further induced the expression of *UGT1A1* significantly (*p* < 0.05 vs. Phe) in the presence of Phe, whereas HS alone treatment (1.08-fold) showed no significant change when compared to control (Figure 8).

**Figure 9:**
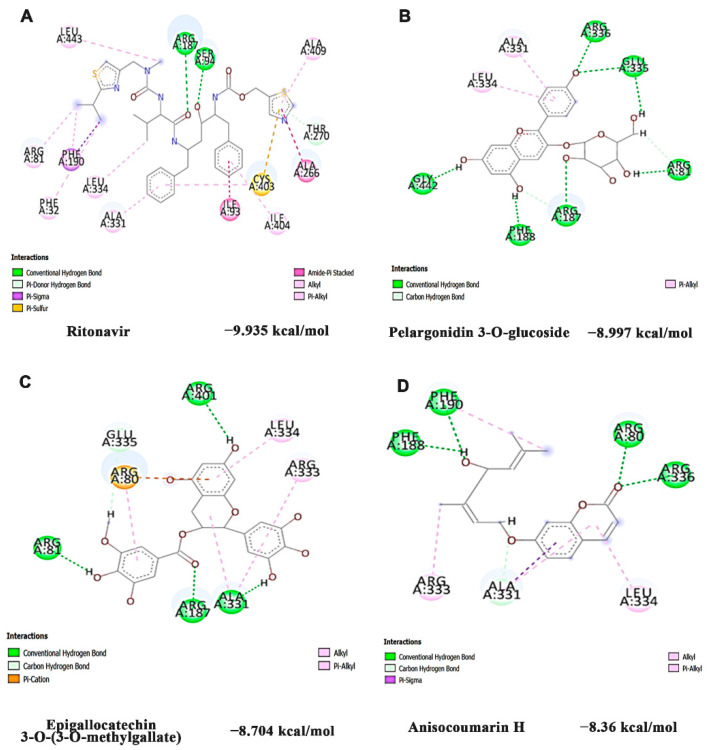
Bioactive constituents of HS extract inhibited CYP3A4 in a site-specific manner as identified by docking analysis (**A**) Ritonavir (antagonist) (**B**) Pelargonidin 3-*O*-glucoside (**C**) Epigallocatechin 3-*O*-(3-*O*-methyl gallate (**D**) Anisocoumarin H.

Further, the inhibitory effect of CYP3A4 by the phytochemicals in HS extract was identified through docking analysis and the results were compared with Ritonavir (antagonist). Ritonavir exhibited the highest docking score (−9.935 kcal/mol), which is followed by Pelargonidin 3-*O*-glucoside (−8.997 kcal/mol), Epigallocatechin 3-*O*-(3-*O*-methylgallate) (−8.704 kcal/mol) and Anisocoumarin H (−8.36 kcal/mol). The interaction of ritonavir and the top 3 hits were represented in Figure 9A–D. The compounds of HS interacted in the same binding site of ritonavir and shared similar interactions (hydrogen, Alkyl, Pi-Sigma, and Pi-Alkyl bond) with the amino acid residues ARG187, ALA331, ARG333, LEU334, along with additional conventional hydrogen bonds indicating strong binding.

### 3.5. HS Extract Inhibited CYP1A1-Induced ROS Generation upon Phe Toxicity and Restored Mitochondrial Membrane Potential (*ΔΨm*) in HaCaT Cells

**Figure 10:**
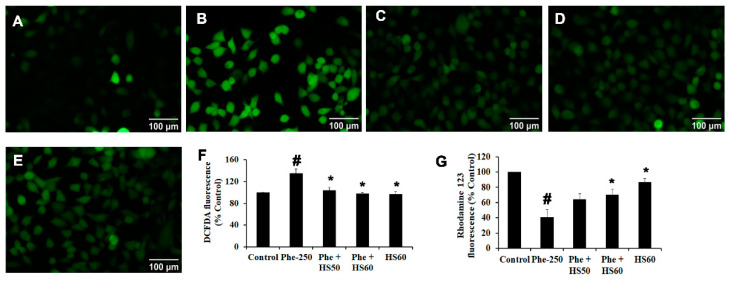
Pre-treatment of HS extract attenuated ROS formation and loss of mitochondrial membrane potential against Phe toxicity. Fluorescence microscopic images representing attenuation of ROS formation by HS (**A**) Control (**B**) Phe (250 μM) (**C**) Phe + HS50 (**D**) Phe + HS60 (**E**) HS60 (**F**) Spectrofluorimetric quantification of ROS generation as assessed by DCFDA assay (**G**) Spectrofluorimetric quantification of mitochondrial membrane potential as assessed by Rhodamine 123 assay (*n* = 3; Significance at *p* < 0.05; # indicates Control vs. Phe; * indicates Phe vs. HS pre-treatment; scale bar—100 μm).

As the activation of CYP1A1 has been reported to induce ROS generation and alter ΔΨm, their changes upon Phe-induced toxicity and pre-treatment with HS were measured. Phe exposure significantly increased ROS formation (135%) compared to the control. However, HS pre-treatment significantly (*p* < 0.05) attenuated ROS generation in a dose-dependent manner when compared to the Phe-exposed group (Figure 10F), indicating the inhibition of oxidative stress and antioxidant potential of HS. The results were also supported by microscopic fluorescence analysis where the increase in green fluorescence was observed in Phe treated group (Figure 10B) compared to the control (Figure 10A), indicating ROS generation. Moreover, HS pre-treatment inhibited the generation of ROS and attenuated the toxicity exerted by Phe (Figure 10C–E). Also, ROS generation induced alterations in mitochondrial architecture, which could be observed from the significant loss of ΔΨm (40.5%) during Phe exposure compared to the control. However, HS extract pre-treatment restored the ΔΨm in a dose-dependent manner indicating the protective effect (Figure 10G).

### 3.6. HS Extract Protected HaCat Cells from Phe-Induced Apoptosis

**Figure 11:**
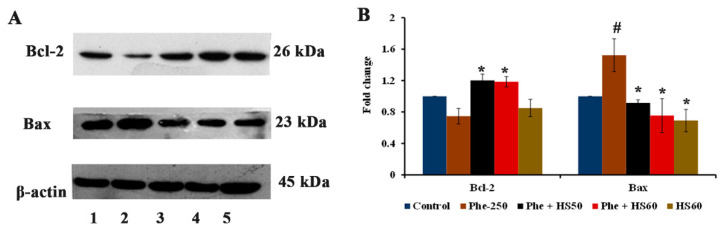
(**A**) Phe-induced apoptosis in HaCaT cells by upregulating Bax expression and downregulating Bcl-2 expression whereas, HS pre-treatment attenuated apoptosis and protected the cells [Lane-1: Control; 2: Phe (250 μM); 3: Phe + HS50; 4: Phe + HS60; 5: HS60] (**B**) Quantification for western blot analysis (*n* = 3; Significance at *p* < 0.05; # indicates Control vs. Phe; * indicates Phe vs. HS pre-treatment).

As ROS generation and alteration of ΔΨm can induce apoptotic cell death, the protein expression of apoptotic and anti-apoptotic proteins Bax and Bcl-2 were evaluated. Phe exposure significantly upregulated the expression of Bax (1.52-fold) and downregulated the expression of Bcl-2 (0.75-fold) compared to that of the control, with Bcl-2 to Bax ratio of 0.49 indicating apoptosis. However, both the concentrations of HS extract restored the changes and exhibited increased Bcl-2 to Bax ratio of 1.31 and 1.57 for 50 and 60 μg/mL, respectively, indicating the inhibition of apoptosis (Figure 11A,B).

### 3.7. HS Extract Regulated the Expression of Stress Response and Gluconeogenesis Related Genes upon Phe Toxicity

**Figure 12:**
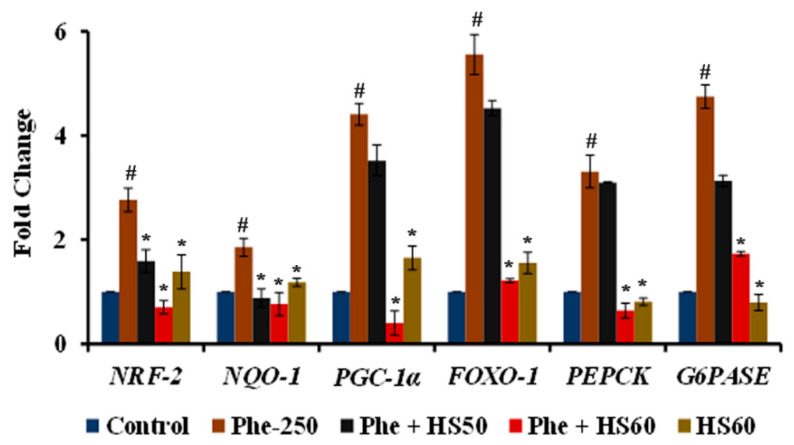
Pre-treatment with HS extract restored the expression of stress response and gluconeogenesis-related genes in HaCaT cells against Phe toxicity (*n* = 3; Significance at *p* < 0.05; # indicates Control vs. Phe; * indicates Phe vs. HS pre-treatment).

A significant (*p* < 0.05) upregulation of stress response genes *NRF-2* (2.76 ± 0.22-fold), *NQO-1* (1.85 ± 0.17), *PGC-1α* (4.41 ± 0.21), and *FOXO-1* (5.55 ± 0.38-fold) was observed upon Phe-induced toxicity in HaCaT cells (Figure 12). Likewise, the gluconeogenesis-related genes *PEPCK* (3.31 ± 0.31-fold) and *G6PASE* (4.75 ± 0.22-fold), which are the downstream target of *CYP3A4,* were also significantly upregulated in response to Phe exposure. Nevertheless, the pre-treatment with HS (60 μg/mL) significantly inhibited the expression of all the genes tested, indicating the attenuation of oxidative stress and gluconeogenesis.

## 4. Discussion

Every day, humans are exposed to environmental pollutants, including PAHs, that may act as carcinogens, teratogens or genotoxicants. Pioneering studies have shown that PAH exposure occurs through inhalation, absorption, and diet [12,13,14,15]. Skin is a major target, as it is highly exposed to environmental pollutants, which cause wrinkling, hyperpigmentation and skin cancer [27,28]. Phe is one of the 16 PAHs which have been described by the EPA as high-priority pollutants [11]. The current study is intended to evaluate the effect of Phe and the mechanism through which it exerts toxicity in HaCaT cells, as well as the ameliorating effect of HS extract against Phe-induced toxicity.

A cross-platform metabolomics study has reported alterations in the amino acid pool and antioxidant mechanism upon Phe exposure in HaCaT cells, indicating the toxic nature of the compound and the need for quenching the effect through suitable antioxidants [50]. Concomitantly, Phe (250 μM) exerted a 50% reduction in cell viability at 24 h through the AhR-independent CYP1A1 activation mechanism. Previous studies have reported Phe as a weak ligand to AhR, and the mediated toxicity is expected to be Ahr-independent [51,52]. While the activation of CYPs plays a major role in detoxifying PAHs entering the body, it is also involved in the generation of toxic metabolites of inert PAHs through oxidation [53]. A recent study by Rusni et al. (2022) reports that Phe was not toxic to *Javanese medaka* with CYP1A1 knockout, with the wild-type being more sensitive, indicating that the biotransformed metabolites of Phe by CYP1A1 are highly toxic [10]. Hence, inhibitors of CYP1A1 can exhibit a protective effect against Phe-induced toxicity. Our results show that Phe-induced CYP1A1 expression (both gene and protein) was significantly inhibited by HS. The results were supported by docking analysis, where the compounds present in HS extract, including Epigallocatechin 3-*O*-(3-*O*-methylgallate), Pelargonidin 3-*O*-glucoside, and *N*-Feruloyltyramine bind on the active site and showed potent inhibitory effect (higher binding scores) against CYP1A1, when compared with the antagonist α-Naphthoflavone [54]. In addition to CYP1A1, Phe-induced gene expression of other *CYPs,* including *CYP1B1* and *CYP2B,* were downregulated upon pre-treatment with HS extract, indicating its protective effect. 

As the experimental results point toward the AhR-independent activation of CYP1A1, the expression of other xenobiotic sensors CAR, PXR, and RXR, which belong to nuclear receptor superfamilies and are capable of inducing CYPs, was analyzed to elucidate the mechanism through which the activation occurs. Following the binding of a suitable ligand, the nuclear receptors, CAR and PXR, form a heterodimer with RXR and bind to their specific response elements resulting in the transcriptional activation of target genes, including those involved in drug and energy metabolism [55]. Apart from the specific activation of CYP3A4, CAR/PXR could also induce CYP1A1 expression [56]. The gene expression results indicate that upon Phe-induced toxicity, the expression of *CAR*, *PXR*, and *RXR* is significantly upregulated along with the induction of *CYP1A1* and *CYP3A4*. Our results are in accordance with the previous study, which reports that Phe can effectively activate CAR to induce hepatotoxicity in mice [57]. The results obtained in vitro were supported with docking analysis, where Phe was found to bind on the active site of both CAR and PXR with similar interaction sites as that of the agonist CITCO [58,59], indicating the activation of the pathways upon Phe exposure. However, HS extract exhibited a protective effect by significantly downregulating the expression of *CAR*, *PXR*, and *RXR,* as well as their target *CYP3A4*. Moreover, the compounds of HS exhibited strong inhibitory potential against CAR (pelargonidin 3-*O*-glucoside, allo aromadendrene, and Epigallocatechin 3-*O*-(3-*O*-methyl gallate) and PXR (Epigallocatechin 3-*O*-(3-*O*-methyl gallate, pelargonidin 3-*O*-glucoside, and anisocoumarin H) similar to the reported inhibitors CINPA1 (CAR) [60] and ketoconazole (PXR) [61]. The interacting site and the amino residues in both the receptors by the compounds were similar to that of Phe. This suggests that the compounds of HS can positively compete with Phe to bind to the receptors and exert their inhibitory effect. Further, the compounds of HS (Pelargonidin 3-*O*-glucoside, Epigallocatechin 3-*O*-(3-*O*-methylgallate) and Anisocoumarin H) showed strong inhibitory potential in docking analysis against CYP3A4 similar to the antagonist Ritonavir [62]. Previous studies have reported the inhibition of CYP3A4 by pelargonidin, green tea catechins and coumarins, indicating the need for exploring the bioactive compounds for their protective activity against Phe exposure [63,64,65].

Pioneering studies have reported on the altered expression and activity of Phase II metabolizing genes and enzymes upon Phe exposure. Epoxide hydrolase 1 (EPHX1) is an important biotransformation enzyme during Phe metabolism. After the initial metabolism by CYPs, EPHX1 acts on the formed metabolites of Phe to convert them into dihydrodiols [66]. Although the metabolism by EPHX1 seems to be more of a detoxification step, the further formed metabolites, including 1-hydroxy phenanthrene and 9-hydroxy phenanthrene, were reported to be toxic in *Danio rerio* [67]. Corroborating with these reports, Phe exposure induced toxicity through the upregulation of *EPHX1*, which is attenuated upon HS extract. The genes *GSTM1* and *GSTP1* encode for the isozymes glutathione S-transferase Mu 1 and Pi 1, respectively. The GSTs catalyze the conjugation of glutathione with the formed electrophilic metabolites, and the reduced expression of GSTs has been reported to limit the detoxification efficiency [68]. Studies on human subjects report that in GSTM1 negative individuals, Phe may enter into bioactivation pathways with higher PAH-DNA adduct formation [69,70]. Sulfotransferase (SULT1A1) and uridine 5′-diphospho-glucuronosyltransferase (UGT1A1) show substrate specificity to phenolic and lipophilic compounds, respectively, thereby enhancing the water solubility for clearance [71], and the expression of both *SULT1A1* and *UGT1A1* are upregulated during Phe exposure [57,72]. In accordance with the previous reports, in the current study, the expression of *GSTM1*, *GSTP1*, *SULT1A1* and *UGT1A1* were significantly upregulated upon Phe exposure, indicating the activation of the host cell’s survival mechanism against toxicity. Meanwhile, HS treatment, except for *UGT1A1*, showed a dose-dependent reduction in the expression of *GSTM1*, *GSTP1*, and *SULT1A1*, which may be because of the unavailability of electrophilic/phenolic metabolites due to the inhibition of CYP1A1 by the extract. 

Additionally, PAH-induced CYP1A1 has been reported to generate ROS, demonstrating a link between xenobiotic mechanisms and oxidative stress [73,74]. With no exception, Phe has been previously demonstrated to induce ROS generation in A549 cells and *Eisenia foetida* and alter ΔΨm in *Gobiocypris rarus* [75,76,77]. In line with the previous reports, Phe exposure to HaCaT cells caused oxidative stress-mediated toxicity and reduced ΔΨm. In addition, oxidative stress upon Phe toxicity induced apoptosis in HaCaT cells, which could be observed from the decrease in Bcl-2 to Bax ratio concomitant to the previous study, where Phe has been reported to induce apoptosis in neuronal cells [36]. However, HS pre-treatment protected the cells from apoptosis by increasing the expression of Bcl-2. Moreover, there was a significant upregulation in the expression of antioxidant response genes *NRF-2*, *NQO-1*, *FOXO-1*, and *PGC-1α* upon exposure to Phe, along with genes involved in gluconeogenesis (*PEPCK*, *G6PASE*). Previous studies have demonstrated that Phe exposure induces the antioxidant response genes to cope with the building toxicity in the host system [76]. However, HS pre-treatment significantly downregulated the expression of stress response genes (*NRF-2*, *NQO-1*, *FOXO-1*, and *PGC-1α*), which could be due to the inhibition of CYP1A1 and associated toxicity. Moreover, treatment with HS alone (60 μg/mL) significantly upregulated the antioxidant genes, which could be due to the reported antioxidant potential of the extract [44]. The transcriptional regulation of *PEPCK* and *G6PASE* is under the control of CAR/PXR/RXR. The activation of the CAR/PXR pathway has been demonstrated to inhibit gluconeogenesis through the suppression of PEPCK and G6PASE [78]. In addition, the transcriptional regulation of *PEPCK* and *G6PASE* is also highly dependent on FOXO-1, the suppression of which subsequently downregulates both genes, thereby affecting gluconeogenesis [79]. Further, PGC-1α acts as a transcriptional co-activator for FOXO-1 and induces gluconeogenesis-related genes [80]. Due to the activation of the *CAR/PXR* pathway, although it is expected for the downregulation of *PEPCK* and *G6PASE*, our study indicated upregulation upon Phe exposure, which is in contradiction with the previous report. This may be regarded as an adaptive strategy by the cells due to the increased need for energy demand, so that glucose can be conserved for ATP generation or channelled towards the phosphogluconate pathway for the generation of NADPH to combat Phe-induced oxidative stress [81,82]. However, HS pre-treatment (60 μg/mL) significantly downregulated the expression of *PEPCK* and *G6PASE* (though expected to be upregulated due to *CAR/PXR* inhibition), which could be due to the downregulation of the co-activators *FOXO-1* and *PGC-1α* upon the attenuation of oxidative stress by the extract to bring cellular homeostasis [83,84]. In addition, calyxes of HS were reported to inhibit gluconeogenesis in older women through the downregulation of the cortisol pathway [85]. Cortisol can stimulate the expression of *PEPCK* and *G6PASE* to induce gluconeogenesis [86]. The downregulation of *PEPCK* and *G6PASE* in the current study could be extrapolated to the attenuation of cortisol by HS extract. 

## 5. Conclusions

The results of our study demonstrate that Phe induces toxicity through CAR/PXR/RXR-mediated CYP1A1 and CYP3A4 activation. Pre-treatment with HS extract effectively inhibited the mechanism and restored the subsequent changes in the transcriptional activity of genes involved in stress response, gluconeogenesis and phase I and II metabolism and attenuated apoptosis in HaCaT cells. The bioactive compounds (epigallocatechin 3-*O*-(3-*O*-methylgallate), pelargonidin 3-*O*-glucoside, anisocoumarin H and *N*-Feruloyltyramine of HS are of potential interest as they could inhibit CYP1A1 and CYP3A4 evidenced by docking analysis. Further studies of the extract and phytochemicals in higher experimental models would provide valuable information on the therapeutic application of *H. sabdariffa* against PAHs.

## Figures and Tables

**Table 1:** List of genes and sequences of primers used for Real-Time PCR analysis.

Sl. No.	Gene Name	Forward Primer	Reverse Primer
1	*AhR*	GTCGTCTAAGGTGTCTGCTGGA	CGCAAACAAAGCCAACTGAGGTG
2	*CYP1A1*	GATTGAGCACTGTCAGGAGAAGC	ATGAGGCTCCAGGAGATAGCAG
3	*CYP1B1*	GCCACTATCACTGACATCTTCGG	CACGACCTGATCCAATTCTGCC
4	*CYP2B*	ACAGTGTGGAGAAGCACCGTGA	GGTTGAGGTTCTGGTGGCTGAA
5	*CAR*	GCAGAAGTGCTTAGATGCTGGC	GCTCCTTACTCAGTTGCACAGG
6	*PXR*	GCTGTCCTACTGCTTGGAAGAC	CTGCATCAGCACATACTCCTCC
7	*RXR*	TTCTCCACCCAGGTGAACTC	GAGCTGATGACCGAGAAAGG
8	*CYP3A4*	CAAGACCCCTTTGTGGAAAA	CGAGGCGACTTTCTTTCATC
9	*UGT1A1*	GCAAAGCGCATGGAGACTAAGG	GGTCCTTGTGAAGGCTGGAGAG
10	*SULT1*	GCAACGCAAAGGATGTGGCA	TCCGTAGGACACTTCTCCGA
11	*GSTP1*	TGGACATGGTGAATGACGGCGT	GGTCTCAAAAGGCTTCAGTTGCC
12	*GSTM1*	TGATGTCCTTGACCTCCACCGT	GCTGGACTTCATGTAGGCAGAG
13	*EPHX1*	GTTTTCCACCTGGACCAATACGG	TGGTGCCTGTTGTCCAGTAGAG
14	*PEPCK*	GTGGGGGATGATATTGCTTG	TGGTCTCAGCCACATTGGTA
15	*G6PASE*	GGGAAAGATAAAGCCGACCTAC	CAGCAAGGTAGATTCGTGACAG
16	*NRF-2*	CACATCCAGTCAGAAACCAGTGG	GGAATGTCTGCGCCAAAAGCTG
17	*NQO-1*	CCTGCCATTCTGAAAGGCTGGT	GTGGTGATGGAAAGCACTGCCT
18	*PGC-1α*	CCAAAGGATGCGCTCTCGTTCA	CGGTGTCTGTAGTGGCTTGACT
19	*FOXO-1*	CTACGAGTGGATGGTCAAGAGC	CCAGTTCCTTCATTCTGCACACG
20	*GAPDH*	GTCTCCTCTGACTTCAACAGCG	ACCACCCTGTTGCTGTAGCCAA

**Table 2:** Grid box parameters for the protein targets.

Grid Box Center	Grid Box Size	CYP1A1 ^1^	CYP3A4 ^2^	CAR ^3^	PXR ^4^
*X*	20	−29.0937	22.5109	25.6018	12.2592
*Y*	20	83.4721	33.3657	54.1912	31.7877
*Z*	20	2.7617	138.7003	29.5870	24.5412

^1^ Cytochrome P450 1A1; ^2^ Cytochrome P450 3A4; ^3^ Constitutive androstane receptor; ^4^ Pregnane X receptor.

## Data Availability

Not applicable.
